# Antiretroviral drugs and acute pancreatitis in HIV/AIDS patients: is there any association? A literature review

**DOI:** 10.1590/S1679-45082014RW2561

**Published:** 2014

**Authors:** Natalia Mejias Oliveira, Felipe Augusto Yamauti Ferreira, Raquel Yumi Yonamine, Ethel Zimberg Chehter

**Affiliations:** 1 Faculdade de Medicina do ABC, Santo André, SP, Brazil

**Keywords:** Pancreatitis/complications, Pancreatitis/etiology, Pancreatitis/epidemiology, Antiretroviral therapy, highly active/methods, Antiretroviral therapy, highly active/adverse effects, HIV, Acquired immunodeficiency syndrome

## Abstract

In HIV-seropositive individuals, the incidence of acute pancreatitis may achieve 40% per year, higher than the 2% found in the general population. Since 1996, when combined antiretroviral therapy, known as HAART (highly active antiretroviral therapy), was introduced, a broad spectrum of harmful factors to the pancreas, such as opportunistic infections and drugs used for chemoprophylaxis, dropped considerably. Nucleotide analogues and metabolic abnormalities, hepatic steatosis and lactic acidosis have emerged as new conditions that can affect the pancreas. To evaluate the role of antiretroviral drugs to treat HIV/AIDS in a scenario of high incidence of acute pancreatitis in this population, a systematic review was performed, including original articles, case reports and case series studies, whose targets were HIV-seropositive patients that developed acute pancreatitis after exposure to any antiretroviral drugs. This association was confirmed after exclusion of other possible etiologies and/or a recurrent episode of acute pancreatitis after re-exposure to the suspected drug. Zidovudine, efavirenz, and protease inhibitors are thought to lead to acute pancreatitis secondary to hyperlipidemia. Nucleotide reverse transcriptase inhibitors, despite being powerful inhibitors of viral replication, induce a wide spectrum of side effects, including myelotoxicity and acute pancreatitis. Didanosine, zalcitabine and stavudine have been reported as causes of acute and chronic pancreatitis. They pose a high risk with cumulative doses. Didanosine with hydroxyurea, alcohol or pentamidine are additional risk factors, leading to lethal pancreatitis, which is not a frequent event. In addition, other drugs used for prophylaxis of AIDS-related opportunistic diseases, such as sulfamethoxazole-trimethoprim and pentamidine, can produce necrotizing pancreatitis. Despite comorbidities that can lead to pancreatic involvement in the HIV/AIDS population, antiretroviral drug-induced pancreatitis should always be considered in the diagnosis of patients with abdominal pain and elevated pancreatic enzymes.

## RESUMO

Em HIV-soropositivos, a incidência de pancreatite aguda pode chegar até 40% ao ano, o que é consideravelmente maior que na população geral, cuja incidência é de 2%. A partir de 1996, com a introdução da terapia antirretroviral combinada, conhecida pela sigla HAART (*highly active antiretroviral therapy*), o espectro de fatores nocivos ao pâncreas, como infecções oportunistas e uso de drogas para sua quimioprofilaxia, diminuiu consideravelmente. Análogos nucleotídeos e anormalidades metabólicas, esteatose hepática e acidose láctica despontaram como novas condições que podem acometer o pâncreas. A fim de avaliar o papel das drogas antirretrovirais para tratamento do HIV/AIDS na incidência elevada de pancreatite aguda nessa população, foi realizada revisão sistemática, com inclusão de artigos originais, relatos e séries de caso, cujos alvos de estudo eram pacientes HIV-soropositivos que evoluíram com pancreatite aguda após exposição a alguma das drogas que compõem o esquema antirretroviral. Essa associação foi confirmada após exclusão de outras possíveis etiologias e/ou recorrência do episódio de pancreatite aguda após reexposição ao fármaco suspeito. Zidovudina, efavirenz e os inibidores de protease são suspeitos de levar a uma pancreatite secundária à hiperlipidemia. Já os análogos nucleotídeos da transcriptase reversa, apesar de serem potentes inibidores da replicação viral, possuem grande espectro de efeitos colaterais, entre eles a mielotoxicidade e a pancreatite aguda. Didanosina, zalcitabina e estavudina já foram reportados como produtores de pancreatite crônica e aguda, tendo risco elevado com dose cumulativa. Didanosina com hidroxiureia, álcool ou pentamidina são fatores de risco adicionais, podendo induzir a uma pancreatite fatal, embora pouco frequente. Além disso, outras drogas usadas para profilaxia de doenças oportunistas relacionadas à AIDS, como sulfametoxazol-trimetoprima e pentamidina, podem produzir pancreatite necrotizante. Apesar das comorbidades que podem levar ao acometimento pancreático na população com HIV/AIDS, pancreatite medicamentosa desencadeada por drogas antirretrovirais sempre deve ser considerada no diagnóstico diferencial desses pacientes que se apresentam com dor abdominal e elevação das enzimas pancreáticas.

## INTRODUCTION

The human immunodeficiency virus (HIV) infection and the acquired immunodeficiency syndrome (AIDS) are worldwide public health problems. The implementation of combined antiretroviral treatment, known by the abbrevaition HAART - highly active antiretroviral therapy, for the treatment of HIV/AIDS, made the remission of the HIV-1 virus possible for long periods, improving the quality of life of these individuals and promoting the fall in HIV virus-related complications and deaths.^(1)^

Notwithstanding, long-term antiretroviral drug-based treatments cause serious toxic effects. The incidence of acute pancreatitis (AP) may reach up to 40% of HIVseropositive individuals a year, which is considerably higher than for the general population, that has an incidence of 2%.^(2)^

Since the HIV is probably directly toxic to the pancreas, pancreatic involvement in patients with AIDS was a common finding in post-mortem studies in the pre-HAART era.^(3–8)^ Chehter et al.^(9)^ found atrophy of acinar cells in 60% of samples from 109 post-mortem pancreas, in addition to decrease in zymogen granules (52%) and nucleus changes (64%), the question remaining if these changes were caused by the HIV virus or by malnutrition, and if they were really capable of leading to pancreas dysfunction.

Moreover, Cappell and Marks^(10)^ showed that 25% of HIV-seropositive patients submitted to abdominal ultrasound and 33% of those submitted to CT scans had pancreas abnormalities, including focal or diffuse enlargements of the organ, dilation of the pancreatic duct, pseudocyst and abscess.

In the pre-HAART era, pentamidine and didanosine, drugs extensively used for AIDS patients, were associated with increased incidence of AP,^(8,11–19)^ mainly when there was a previous episode of AP, prolonged treatment with high doses and severe immunocompromised conditions.^(20)^ In the Cappell and Marks series,^(10)^ among 18 patients with drug-related AP, pentamidine was responsible for 12 cases, followed by didanosine with 4, and sulfamethoxazole-trimethoprim with 2 cases. When compared to the control group, these patients were younger (mean age of 35.2 years *versus* 49.1 years) and, generally, men (77% *versus* 48%) and black individuals (77% *versus* 11%).

Despite the evidence, HIV/AIDS patients may present a wide range of toxic factors to the pancreas, such as opportunistic infections by *Pneumocystis jiroveci* and *Mycobacterium avium* complex, neoplasms and metabolic changes due to use of antiretroviral drugs. With the introduction of HAART and consequent reduction in the need for treatment and chemoprophylaxis for opportunistic infections, the administration of combined antiretroviral agents and metabolic abnormalities, such as liver steatosis and lactic acidosis, emerged as new conditions affecting the pancreas.^(21,22)^ Thus, acknowledging that: drug induced AP occurs after beginning treatment with a certain drug; that the resolution of the clinical picture occurs with withdrawal of treatment; and that the condition returns with the re-administration of medication, without other apparent causes of pancreatitis;^(23)^ the objective of the present study was to assess the role of the most common antiretroviral drugs used to treat HIV/AIDS individuals in the development of episodes of AP, mainly after 1996, when the HAART regimen began to be used routinely.

## METHODS

The literature review was systematic, searching publications in Portuguese, English and Spanish in the following databases, from the emergence of the condition to February 2012: MEDLINE (from 1990 to 2012); LILACS (from 1983 to 2012) and Cochrane Library (from 1993 to 2012). The keywords used for the study were: “*pancreatite aguda*/acute pancreatitis”; “HIV - human immunodeficiency virus”; “AIDS -acquired immunodeficiency syndrome”; and “*Terapia antirretroviral de alta atividade*/HAART – highly active antiretroviral therapy”.

The authors included studies based on the title and abstract. When studies were classified as eligible, a copy of the entire article was requested, to apply inclusion criteria. The study was considered non-eligible, when it was not the subject of interest and/or it met exclusion criteria. The authors collected data, such as study population and associated risk factors, exposure or not to antiretrovirals, definition of AP used by the authors and, finally, the conclusion of the study on the relation between AP and the HAART regimen. Then, the methodological quality was assessed and results were analyzed independently. In case of disagreements, the classification of the studies was discussed in a meeting to establish a consensus among authors.

Thus, the authors selected original articles, reports and case series that aimed to study HIV-positive patients that developed AP after exposure to any of the drugs in the HAART regimen, and that had this association confirmed after ruling out other possible etiologies and/or recurrence of the AP episode after re-exposure to the suspected drug.

Abdominal pain associated with high pancreatic enzymes (amylase and lipase) three times above the upper normal limit, and abnormalities observed on the ultrasound and/or CT scan were considered AP.

All drugs comprising any HIV/AIDS treatment regimen were also considered: nucleotide reverse-transcriptase inhibitors (NRTI), non-nucleoside reverse-transcriptase inhibitors (NNRTI) and proteases inhibitors (PI).

Articles not published in portuguese, english and spanish; articles that did not explicit diagnostic criteria of AP; studies conducted exclusively before 1996 when the concept of combined antiretroviral therapy (HAART) was introduced; and articles that only related HIV/AIDS with non-specific pancreatic alterations were excluded.

The quality of studies was assessed using a Delphi list, using nine questions with three possible answers (yes, no and do not know), as internal and external validity, and statistical considerations.

## RESULTS

We identified 64 articles, 23 of which met selection criteria. The studies included in the review are represented in charts 1 and 2.

**Chart 1:** Association of antirretroviral drugs, comorbidities and acute pancreatitis in selected case reports

Author/year/originm	Antirretroviral regimen	Comorbidities/ associated conditions	Re-exposure to a suspected drug (yes/no)	Is AP associated to HAART?
Allaouchiche et al.^(24)^, 1999, France	Didanosine, stavudine, indinavir	Kaposi sarcoma, lactic acidosis	No	No, presence of other conditions
Sarner & Fakoya^(25)^, 2002, England	Stavudine, didanosine, nevirapine (2 cases)	Case 1: pregnant woman 37 weeks, HELLP and treated tuberculosis	No	Case 1: No, presence of lactic acidosis and HELLP
		Case 2: pregnant woman 33 weeks		Case 2: yes
Kirian et al.^(26)^, 2004, United States	Didanosine (low dosage), tenofovir, lamivudine, stavudine, efavirenz	Hypertriglyceridemia, diabetes mellitus, prophylaxis of opportunistic infections	No	Yes, probably association of tenofovir and didanosine
Blanchard et al.^(27)^, 2003, United States	Didanosin and tenofovir in common + other antirretroviral (4 cases)	Low CD4 count, associated opportunistic infections, cholelithiasis, hyperlipidemia, marked weight loss	No	Yes, tenofovir and didanosine
Callens et al.^(28)^, 2003, Belgium	Zidovudine, ritonavir	No description	No	Yes
Longhurst & Pinching^(29)^, 2001, England	Estavudina, didanosina, nevirapina	Association with hydroxyurea	No	Yes, ritonavir due to induction of hypertriglyceridemia
Mirete et al.^(30)^, 1998, Colombia	Zidovudina, ritonavir	No description	No	Sim
Perry et al.^(31)^, 1999, United States	Ritonavir, saquinavir, zidovudine, lamivudine, delaviridine	Hypertriglyceridemia	Yes	Yes, ritonavir due to induction of hypertriglyceridemia
Di Martino et al.^(32)^, 1999, France	Zidovudine, nelfinavir	No description	Yes	Yes
Chapman et al.^(33)^, 2007, England	Tenofovir, zidovudine, lamivudine, abacavir, tipranavir, ritonavir	Alcohol abuse, associated opportunistic infections	No	Yes, tipranavir and ritonavir due to induction of hypertriglyceridemia
Trindade et al.^(34)^, 2008, United States	Abacavir, lamivudine	Familiar hypertriglyceridemia, estrogen therapy, prophylaxis of opportunistic infections	No	No, presence of other conditions that led to AP

AP: acute pancreatitis, HAART: highly active antiretroviral therapy.

**Chart 2:** Characteristics, mains risk factors and antiretroviral drugs related to acute pancreatitis in selected studies

Author/year/origin	Type of study/period	n	Etilogy/risk factors	Main antiretroviral associate dto AP	Is AP associated to HAART?
Anderson et al.^(35)^, 2008, South Africa	Prospective cohort (2001-2006)	282	Alcohol (62%), biliary (14%), dyslipidemia (8%), antiretroviral agents (5%)	Didanosine, stavudine	Yes
Moore et al.^(36)^, 2001, United States	Prospective	2,613 (33 cases of AP)	Hydroxyurea, female gender, past history of AP, CD4 count <200 cells/mm^3^	Didanosine with hydroxyurea	Yes
Gan et al.^(37)^, 2003, Canada	Prospective	73	AIDS, severe immunosuppression	Didanosine in different regimens	Yes; 46% of AP due to drugs; polypharmacy
Manfredi et al.^(38)^, 2004, Italy	Case control	920 (128 cases of AP)	Alcohol, opportunistic infections and prophylaxis, liver or biliary diseases, PI, hypertriglyceridemia	Didanosine, stavudine, lamivudine, PI	Yes
Riedel et al.^(39)^, 2008, United States	Case control (1996-2006)	5,970 (85 cases of AP)	Females, CD4 count <50 cells/mm^3^, stavudine and pentamidine	Stavudine	Yes
Guo et al.^(40)^, 2005, United States	Retrospective (1997-2002)	4,972 (159 cases of AP)	Non-Caucasian, advanced age, AIDS, liver and cardiovascular diseases	Didanosine with NRTI or NNRTI or PI	No
Reisler et al.^(41)^, 2006, United States	20 clinical studies (1989-1999)	8,451(incidence of AP of 2.23 cases/100)	-	Didanosine, stavudine and indinavir with or without hydroxyurea	Yes
Smith et al.^(42)^, 2008, England	Retrospective (2001-2006)	9,678 (43 cases of AP)	Low CD4 count	Didanosine, stavudine	No
Barrios et al.^(43)^, 2004, United States	Prospective	309	-	Didanosine, tenofovir, efavirenz	No; there were no cases of AP or neuropathy
Martinez et al.^(44)^, 2004, Spain	Retrospective (2001-2003)	575 (6 cases of AP)	-	Didanosine and tenofovir	Yes; greater incidence when associated
Bush et al.^(45)^, 2003, United States	Retrospective (1990-2001)	250 (84 cases of AP)	Alcohol, opportunistic infections	Didanosine, stavudine, lamivudine, zalcitabine, PI	Incidence of AP did not change after PI introduction
Manfredi e Calza^(46)^, 2008, Italy	Prospective cohort (2005-2006)	1,081	Time of HIV infection, AIDS, hepatobiliary disease, hypertriglyceridemia, alcohol, illicit drugs, prophylaxis of opportunistic infections	Didanosine, stavudine, lamivudine, PI	Yes

AP: acute pancreatitis. HAART: highly active antiretroviral therapy; PI: protease inhibitors; NRTI: nucleoside reverse transcriptase inhibitors; NNRTI: non- nucleoside reverse transcriptase inhibitors.

After 1996, there was an increase in cases of AP attributed to drugs, mainly to combined antiretroviral therapy and not to a specific drug. A study from South Africa^(35)^ found an incidence of 5% for antiretroviral-related AP, especially didanosine and stavudine. However, like in the general population, the main cause of AP remained alcohol abuse, followed by AP of biliary origin.

Trivedi et al.^(2)^ selected the one hundred most prescribed drugs by physicians in the United States, evaluating their association with AP. Didanosine, with 883 cases reported and 9 cases after re-exposure, was the main drug associated with AP. Moreover, in class I (at least 20 AP cases reported), we found pentamidine and sulfamethoxazole-trimethoprim. In class II (between 10 and 20 reports of AP), was lamivudine, while in class III (at least 1 case of AP), were most of the drugs used in the HAART regimen, such as abacavir, stavudine, zidovudine, indinavir, ritonavir, efavirenz, among others.

Didanosine, alone or in different combinations (didanosine/stavudine/indinavir; didanosine/stavudine/nevirapine; didanosine/tenofovir), was strongly associated with medication-induced AP.^(24–29,47)^

A study conducted by Moore et al.,^(36)^ analyzed 2,613 patients on treatment with 6 different NRTI regimens. The incidence of AP was lower among those who used only zidovudine, didanosine or stavudine, while the group on didanosine associated with hydroxyurea, to boost action, had an increased risk, possibly because of increased mitochondrial dysfunction. On the other hand, the concomitant use of PI or NNRTI did not increase the risk for AP.

Gan et al.,^(37)^ analyzed 73 men infected by the HIV virus that developed AP, between 1989 and 1999, and observed that the most frequent etiology (46%) was drug related, mainly with didanosine and pentamidine. A peak in the incidence of AP in the mid 1990′s, with a trend towards a fall in the following years, probably represents a change in the practice of prescribing didanosine.

After the introduction of HAART, Manfredi et al.^(38)^ expected changes in HIV-related pancreatic abnormalities. Among 334 patients with a single episode of laboratory abnormal pancreatic enzymes, no relation was established between the duration of the administration of nucleoside analogues and AP. However, prolonged abnormal pancreatic enzymes, with or without clinical manifestations, occurred in 128 patients, and were attributed to the ongoing administration of didanosine, stavudine, PI, pentamidine, lamivudine, sulfamethoxazoletrimethoprim or anti-tuberculosis therapy, alcohol abuse, opportunistic infections, chronic hepatobiliary disease and hypertriglyceridemia.

According to a study performed by Riedel et al.,^(39)^ at the Johns Hopkins Hospital, between 1996 and 2006, the incidence of AP that required admission to hospital was ten-fold higher among HIV patients than in the general population. However, there were no significant differences between incidence of AP in the pre and post-HAART eras, with a slighter lower number of hospitalizations in the HAART era attributed to less use of some NRTI (didanosine and stavudine) and hydroxyurea. PI or NNRTI were not associated with increased rates of AP, as the most recent antiretroviral drugs (atazanavir, ritonavir, tenofovir, abacavir or efavirenz).^(39)^

Guo et al.,^(40)^ on the other hand, after a retrospective study of 4972 patients with HIV infection, found 159 cases of AP, most of which in patients who had begun treatment recently. However, the risk did not change for patients on different treatment antiretroviral regimens, including use of didanosine.

In a study with about 3000 patients, the combination of selected NRTI impacted on the incidence of AP (0.85/100 persons/year).^(41)^ Despite major differences in several arms of the study, didanosine/stavudine was observed to be associated with the highest rates of the condition. In protocols that included PI, combining nevirapine or indinavir with nucleosides, the incidence of AP was similar to groups that used exclusively nucleosides. In this scenario, the association indinavir/didanosine/stavudine was the regimen that resulted in the highest rates of pancreatitis due inducing metabolic and lipid alterations at the cell level, in addition to leading to microlithiasis, resulting in biliary pancreatitis.

The presence of hydroxyurea did not significantly change pancreatitis rates, having been similar for patients on didanosine/hydroxyurea and only on didanosine. In contrast, the frequency of didanosineinduced AP seemed to dose-dependent and related to its high plasma concentration, mainly when taken along with tenofovir^(41)^.

The multicenter EuroSIDA^(42)^ study observed a low incidence of pancreatitis among patients with HIV/AIDS. Although a great part of these patients had contact with NRTI at some point before developing AP, the investigators did not find a relation of the disease with a specific antiretroviral drug or combination of them.

Another two studies tried to assess safety and efficacy of administering didanosine and tenofovir. In the first study, 309 patients had their antiretroviral regimen replaced by didanosine, tenofovir and efavirenz. After 6 months, no event of AP or neuropathy was observed.^(43)^ In the second study, 185 patients were studied after the prescription of didanosine (250 or 400mg), tenofovir and a third unspecified drug. Five female low-body weight patients (47-56kg) developed AP after a mean of 22 weeks of treatment.^(44)^ The study also concluded that lower doses of didanosine, when associated with tenofovir, may help keep safe therapeutic levels, without increasing morbidity.

Similar to Riedel et al.^(39)^ and Reisler et al.^(41)^, Bush and Kosmiski^(45)^ pointed out that neither PI or NNRTI led to a true increase in the incidence of AP, despite initial fears and reports of PI-induced hypertriglyceridemia.

Another study, performed by Manfredi and Calza^(46)^ observed a high incidence and longer duration of pancreatic abnormalities in patients with long-term PI administration (over 6 months), parallel to existing hypertriglyceridemia. In some cases, the severe picture of AP is attributed to hypertriglyceridemia;^(19,30,31)^ in others, it occurred after re-exposure to the medication, without rise in triglycerides.^(45)^

Chapman^(33)^ reported a case of a patient with previous opportunistic infections and a history of alcohol abuse who developed a hypertriglyceridemia-induced AP and that resolved after interruption of tipranavir and ritonavir. Trindade et al.^(34)^ described a HIV-infected woman with AP, on abacavir and lamivudine, which rarely leads to pancreatic lesion. There was however a family history of hypertriglyceridemia and use of estrogens and sulfamethoxazole-trimethoprim, also involved in episodes of AP.

Table 1 describes the main risk factors associated with AP in patients with HIV/AIDS on antiretroviral treatment.

**Table 1:** Risk factors for acute pancreatitis in HIV/AIDS patients

NRTI	Didanosine
	Stavudine
	Lamivudine
	Tenofovir
	Zalcitabine
	Zidovudine
NNRTI	Efavirenz
	Nevirapine
PI – induction of hypertriglyceridemia	Ritonavir
	Tipranavir
	Indinavir
	Nelfinavir
Direct lesion by HIV	
Diagnosis of AIDS/opportunistic infections and neoplasms	
CD4 count <200 cells/mm^3^	
High viral load	
Alcohol abuse	
Use of illicit drugs	
Past history of acute pancreatitis	
Long seropositivity duration	
Advanced age	
Non Caucasian	
Hepatobiliary diseases	
Liver steatosis and lactic acidosis	
Female and low weight (47-56kg)	
Combination of antiretroviral and hydroxyurea	

NRTI: nucleoside reverse transcriptase inhibitors; NNRTI: non-nucleoside reverse transcriptase inhibitors; PI: protease inhibitors.

## DISCUSSION

The HIV infection becomes chronic if treatment begins in the initial stage and is taken without interruption. However, while antiretroviral treatment is highly efficacious, it is also very complex and dangerous, due to its toxic effects, including drug-induced pancreatitis. There is a wide range of factors that can affect the pancreas, from the direct lesion by the HIV, opportunistic infections and neoplasms, alcohol abuse and use of illicit drugs and antiretroviral drugs. As observed in several studies,^(36,38–40)^ HIV-positive patients that develop AP, in addition to exposure to the HAART regimen drugs, have in common advanced age, non-white race (risk 39 to 54% higher for non-whites compared to Caucasians), long duration of seropositivity, CD4 <200 cells/mm^3^, AIDS diagnosis, high viral load, previous history of AP, hepatobiliary diseases, opportunistic infection prophylaxis, alcohol abuse, in addition to, most of the time, women with a low body mass index (BMI) (more sensitive to toxic effects). That is exactly why it is difficult to evaluate the toxic effect of the HAART regimen on the pancreas.

Despite this, zidovudine, efavirenz and PI are suspected of leading to AP secondary to hyperlipidemia. NRTI, on the other hand, cause a series of side effects – among which myelotoxicity, lactic acidosis, polyneuropathy and AP. Didanosine, zalcitabine and stavudine were reported to lead to chronic and acute pancreatitis, with a high risk of cumulative dose. Didanosine with hydroxyurea, alcohol or pentamidine are additional risk factors, and may induce to lethal pancreatitis.

As part of HAART, PI have led to a significant decline in HIV-related morbidity and mortality.^(48)^ However, PIbased treatments are, in general, associated with changes in the distribution of body fat and with metabolic disorders, such as insulin resistance and hypertriglyceridemia.^(21,49)^ The latter is usually severe and difficult to control, and may trigger episodes of AP. However, it must be taken into account that many patients do not have alternatives to PI, because the infection is resistant to other classes of antiretroviral drugs.^(21)^

Thus, many of the drugs prescribed in offices routinely are suspected of causing AP. However, drug-induced pancreatitis is frequently not acknowledged, because mild cases, with significant increases (but not critical) in amylase and lipase levels, may go unnoticed, in addition to the possible dissociation at the time of exposure to the drug and the development of AP. This condition can also be confounded with alcohol-induced or biliary pancreatitis, and there are no features to differentiate them. Therefore, physicians should be highly suspicious, mainly in patients that use several drugs simultaneously.

In this context, it is essential to acknowledge the adverse effects caused by HAART, aimed at improving tolerability and efficacy of treatment for HIV, promoting the early recognition and reversion of these potentially serious effects, and reducing the potential for adverse drug interactions.

However, more evidence is still necessary to determine if pancreatic morbidity is directly related to drugs used in HAART therapy or to other comorbidities.

## CONCLUSION

Drug-induced pancreatitis triggered by antiretroviral drugs of the HAART regimen should always be considered in the differential diagnosis of HIV/AIDS patients that present with abdominal pain and high levels of pancreatic enzymes.
